# Treatment of Non-Union of Fractures

**Published:** 1886-03

**Authors:** 


### Editorial.

---

### Treatment of Non-Union of Fractures.

In a communication on the treatment of non-union of fractures to the Medical Society of the State of New York, Dr. Fowler, of Brooklyn, advocates the trial of the "percussive method" of Thomas, of Liverpool, in all cases of simple delayed union which do not depend upon some dyscrasia; and in cases of fibrous union, provided absorption and attenuation of the ends of the fragments, or eburnation has not taken place, and particularly in those cases where the neighborhood of a joint increases the probability of the failure of other methods, subcutaneous puncture, injections, etc. The method is intended to take the place of the time-worn practice of rubbing the fractured ends together. "The principal objection to the latter method is to be found in the fact, that we have no means of determining before removal of the splint, at the end of the period allowed for consolidation, whether or not the friction has been sufficient to set up the requisite amount of irritation needful for the purpose of exciting renewed efforts at repair; should this have failed, and there must always remain a certain proportion of cases in which it will fail, much valuable time will have been lost, and the patient will be in a worse condition, as regards the fracture, than when the primary dressings were removed." Thomas' method, as explained by the author, consists in percussing the parts about the seat of fracture by means of a small copper mallet, faced with rubber, for from five to ten minutes at a time, repeating the process at intervals of forty-eight hours or longer, until undoubted evidences are afforded, these being the occurrence of pain, heat and swelling, of a renewal of active hyperæmia, and engorgement of the parts.

In his first case, treated in March, 1874, the non-union had existed for twelve months. No splint whatever was applied during the treatment, and the patient left the hospital cured in about eight weeks. The case was one of ununited fracture of the tibia and fibula, and when the patient attempted to bear any weight upon that limb, an outward luxation of the foot occurred to such an extent that the internal malleolus of the tibia nearly came in contact with the ground, and the fracture threatened to become compound. * * * In his subsequent cases he made use of retentive apparatus, with the result, as a rule, of considerably shortening the period of treatment. The author proceeds to say: Have made use of this treatment in several cases in my hospital service, and in all with the most brilliant results. The advantages must be apparent at a glance. It is perfectly safe, its effects are entirely under the Surgeon's control, and, above all, it is most gratifying in its results. In its application to ununited fractures of the shafts of the bones of the lower extremity, it is better to first apply a plaster of Paris bandage, leaving a large fenestrum at the point where the percussion is to be applied. In the forearm, gutter-shaped splints of molded leather or felt may be made use of, the limb to remain in the same while being percussed and then rebandaged. Fractures of the humerous may be maintained in good position during the intervals by the interposition of a soft cushion filled with curled hair, or a like material between its inner surface and the chest wall. The arm is then confined, by a few turns of a roller, to the chest wall, and the forearm held in a comfortable position by means of a handkerchief sling.

